# Damage Control for Vascular Trauma from the Prehospital to the Operating Room Setting

**DOI:** 10.3389/fsurg.2017.00073

**Published:** 2017-12-19

**Authors:** Emmanouil Pikoulis, Karim M. Salem, Efthymios D. Avgerinos, Anastasia Pikouli, Anastasios Angelou, Antreas Pikoulis, Sotirios Georgopoulos, Ioannis Karavokyros

**Affiliations:** ^1^1st Department of Surgery, Laiko Hospital, School of Medicine, University of Athens, Athens, Greece; ^2^Division of Vascular Surgery, University of Pittsburgh Medical Center, Pittsburgh, PA, United States

**Keywords:** vascular trauma, damage control, prehospital, tourniquets, topical hemostatic agents

## Abstract

Early management of vascular injury, starting at the field, is imperative for survival no less than any operative maneuver. Contemporary prehospital management of vascular trauma, including appropriate fluid and volume infusion, tourniquets, and hemostatic agents, has reversed the historically known limb hemorrhage as a leading cause of death. In this context, damage control (DC) surgery has evolved to DC resuscitation (DCR) as an overarching concept that draws together preoperative and operative interventions aiming at rapidly reducing bleeding from vascular disruption, optimizing oxygenation, and clinical outcomes. This review addresses contemporary DCR techniques from the prehospital to the surgical setting, focusing on civilian vascular injuries.

## Introduction

Trauma damage control (DC) is an established strategy for optimal management of injured patients targeting to minimize morbidity and mortality. It includes DC resuscitation (DCR) and DC surgery. DCR is a new concept that includes rapid hemorrhage control, permissive hypotension, and appropriate fluid administration (1). DC surgery in vascular trauma is most often used to control exsanguinating hemorrhage in high-risk injury patients.

Existing literature estimates that up to 20% of deaths after trauma may be preventable, with the majority of these being due to uncontrolled hemorrhage. Further hemorrhage may occur in 25% of trauma admissions due to ongoing and worsening coagulopathy. The risk of mortality is 3–4 times higher in patients with coagulopathy when compared to those without (2). The concept of DCR based on infusion of blood products was developed by military physicians in the early 2000s. Drastic improvement in post-operative coagulopathy and reduction of edema made it popular among civilian trauma centers as well (3).

Contemporary DC algorithms focus on expedited and focused surgical intervention with concomitant rapid reversal of acidosis, prevention of hypothermia, and prevention of coagulopathy (4).

This review focuses on DCR for civilian vascular trauma from the prehospital to the operating room.

## Fluid Resuscitation

Hemorrhage is an etiologic factor of mortality in 30–40% of trauma patients, and many of these deaths happen before the patient reaches the hospital. Advanced trauma life support recommends the prehospital assessment of a patient’s circulation status and resuscitation with intravenous fluids. Fluid resuscitation is aimed at replacing lost volume to maintain perfusion of vital organs until definitive care (5).

Fluid therapy can be achieved by administering crystalloid solutions, colloidal solutions, or blood products (6).

Aqueous fluids used for resuscitation are termed based on additional elements found within the solutions. Fluids which contain electrolytes (crystal-forming elements) are termed crystalloids, while those which contain electrolytes and large organic molecules are termed colloids.

## Crystalloids

Crystalloid resuscitation occurs when electrolytes pass through the endothelial membrane followed by water. The flow of water from the intravascular space into the extracellular space eventually reaches an equilibrium when concentrations are similar in both spaces. Solutions may contain an assortment of organic anions and inorganic cations. Organic anions include acetate, bicarbonate, gluconate, lactate, or Cl^−^, while K^+^, Ca^++^, and Mg^++^ constitute the commonly used inorganic cations.

Commonly used crystalloids include normal saline (NS), Ringer’s lactate (LR), Hartmann’s solution, and Plasma-Lyte. Despite its nomenclature, NS is not physiologic as an electrolyte solution. The solution contains an equal amount of Na^+^ and Cl^−^ making it hypernatremic, hyperchloremic, and slightly hypertonic. A hypernatremic, hyperchloremic, metabolic acidosis can occur with substantial NS infusions. Patients at risk of morbidity from these metabolic derangements (e.g., compromised renal function) may benefit from an alternative crystalloid solution (7).

Solutions such as Hartmann’s solution, Plasma-Lyte, and LR more closely resemble the electrolyte composition of plasma (Table 1). A key characteristic of these solutions is that they are isotonic relative to blood. Although these solutions are more balanced or physiologic, several randomized trials have failed to prove superiority in outcomes of acute kidney injury, major adverse kidney events, or mortality (8, 9).

**Table 1:** Composition of plasma and commonly used crystalloids.

Fluid	Osmolality (mOsm/kg)	Osmolarity (mOsm/L)	Na^+^	Cl^−^	K^+^	Ca^2+^	Mg^2+^
Plasma	288	291	142	103	4.5	2.5	1.25
0.9% Saline	286	308	154	154	0	0	0
Ringer’s lactate	254	273	130	109	4	2.7	0
Hartmann’s solution	257	276	131	111	5	2	0
Plasma-Lyte	–	295	140	98	5	0	1.5

*Concentration of constituents in millimoles per liter*.

## Colloids

Large organic macromolecules and electrolytes contained within colloid solutions are better maintained within the intravascular space. It is postulated that the large size of the macromolecules limits their ability to cross the endothelial membrane. Water is thus retained within the intravascular space due to the increased intravascular oncotic pressure.

The oldest and most commonly used solution is albumin. Concentrations ranging from 4 to 25% are used clinically. Presently synthetic colloids such as dextran, gelatins, and starches (HES) are increasingly being used due to the varying cost of albumin solutions. Gelatins and albumin exist in hypooncotic concentrations, while dextran, HES, and albumin exist in hyperoncotic solutions (7).

The Saline versus Albumin Fluid Evaluation trial randomized 6,997 patients to receive either 4% albumin or NS resuscitation for 28 days in the intensive care unit (ICU). No significant differences in ICU days, hospital days, mechanical ventilation days, or renal-replacement therapy days were found throughout the study period (8). Similar results have been shown in other studies comparing the two resuscitation modalities (10).

Hemodynamic optimization of critically ill patients has been extensively studied in recent years. Goal-directed therapy protocols based on hemodynamic parameters such as central venous oxygenation, oxygen delivery, stroke volume, or cardiac output are now common within ICUs. Meta-analysis of six trials comprising 3,323 patients failed to prove a statistically significant reduction in mortality when a goal-directed algorithm was followed (11).

## Blood Products

Recent literature has also led to changes in the tenets of hemostatic resuscitation. A “1:1” transfusion ratio of fresh frozen plasma (FFP) and red blood cells (RBCs) has been shown to reduce mortality in military literature (2). Several studies have aimed at evaluating whether a specific FFP:RBC ratio best reduces morbidity and mortality in patients with polytrauma, though, a consensus has yet to be reached (12). There is a growing body of literature which suggests that in patients requiring massive transfusion (MT), increased use of FFP and platelets (PLT) improves survival (13, 14). In this patient population requiring MT, higher FFP:RBC and PLT:RBC ratios were associated with increased 6-h, 24-h, and 30-day survival. Additionally, these strategies were associated with decreased ventilator and ICU days (13, 14). While increased FFP and PLT administration ratios may provide benefit in patients who require MT, euvolemic patients requiring less than 10 units of RBCs do not seem to gain any benefit in survival rates (15, 16).

European guidelines recommend that patients with traumatic injuries should be maintained at target hemoglobin (Hb) levels between 7 and 9 g/dL (17). Several randomized controlled trials (RCTs) have shown that liberal transfusion strategies (Hb ≥ 9 g/dL) are not as safe as restrictive transfusion strategies (Hb 7–9 g/dL) in critically ill patients (18, 19). As of now, no prospective RCTs exist which compare liberal and restrictive transfusion strategies in trauma patients specifically.

Adequate tissue oxygenation remains the mainstay of goal-directed resuscitation. Historically, aggressive fluid resuscitation with a normotensive target [systolic blood pressure (SBP) above 100 mmHg] was used. This commonly resulted in dilution of coagulation factors, hypothermia, and reversal of vasoconstriction, thus leading to the use of a permissive hypotension strategy (goal SBP 50–100 mmHg).

## Tourniquets

Tourniquets have been used to aid in control of vascular injuries since the second century AD. French surgeon Jean-Louis Petit described the modern tourniquet in 1817, followed by Harvey Cushing in 1904, who first described the pneumatic tourniquet. Tourniquet use may be required in two differing situations. Surgical tourniquets are used to maintain a bloodless field during elective surgery, while emergency tourniquets are used in trauma. Pneumatic tourniquets are currently the most commonly used (20).

For the patient with significant extremity hemorrhage, the failure to place a tourniquet and stop bleeding may potentially result in exsanguination within minutes. The best evidence for tourniquet use in the prehospital environment comes from the experience of military hospitals. A prospective study analyzing 428 tourniquets placed on 309 injured limbs showed that early tourniquet use before the onset of shock was associated with a 90% survival rate versus 10% survival if the application was delayed until the casualty was in shock (21).

Retrospective studies have examined the use of prehospital tourniquets in civilian trauma. In a 2007 review of tourniquet use in the prehospital setting, it was found that immediate application of a tourniquet may be justifiable in: (a) life-threatening limb hemorrhage, amputation, or a mangled extremity, (b) life-threatening limb hemorrhage not controlled by simple methods, (c) entrapment of a limb preventing access to a point of hemorrhage, (d) multiple casualties with extremity hemorrhage and inability to perform simple methods of hemorrhage control, or (e) benefits of prevention of death outweigh limb loss from ischemia caused by use of a tourniquet (22).

When studied in the urban emergency medical service (EMS) setting, prehospital tourniquet use appeared to be safe. The Boston EMS experience with prehospital tourniquets reported that 91% (95/98) of cases resulted in successful control of hemorrhage, and they were in place for an average of 14.9 min prior to hospital arrival. Tourniquets were removed in the emergency department in 54.7% (52/95) of cases, and in the operating room in 31.6% (30/95) of cases. Of the 30 tourniquets removed in the operating room 14 did indeed have a documented vascular injury which required repair. A complication rate of 2.1% was reported in that study, showing that effective tourniquet application may be used in the prehospital setting safely (23). Despite this, complications of tourniquet use are well documented including: arterial injury and thrombosis, deep venous thrombosis, and neuronal injury. It has been documented in the literature that up to 65% of tourniquet gauges are inaccurate and inappropriate pressures up to 500 mmHg have been applied to limbs improperly (24). Regardless of these potential complications, the use of tourniquets in the prehospital setting has been shown to be effective and potentially life-saving. Education on proper tourniquet use is a key to the success of this strategy.

Recent initiatives in the United States have aimed at educating citizens to provide bleeding control for those in need. The “Stop the Bleed” campaign encourages citizens to learn how to prevent or slow potentially life-threatening hemorrhage as well as have access to and learn to use bleeding control kits. In a national survey of citizens in the United States only one-half reported first aid training at some point in their lives while only 13% had training within the last 2 years. Approximately 25% of those who had training indicated that it did not include education on control of severe hemorrhage (25). Further education of the general public may enable them to act as immediate responders and improve prehospital care and subsequent patient outcomes.

## Hemostatic Agents

Ideal characteristics for hemostatic dressings were previously described as able to stop severe arterial and/or venous bleeding in under 2 min, be without systemic or local toxicity/side effects, cause no pain or thermal injuries, pose no risk to provider applying the dressing, ready to use, easily packed into wounds, be inexpensive and cost-effective, and have a long shelf life (26). The use of topical hemostatic dressings in the prehospital setting for the control of massive hemorrhage has the potential to alter the current standard of care for exsanguinating patients and improve the clinical outcome in heavy injured patients.

Prior to Operation Enduring Freedom and Operation Iraqi Freedom the Army Field Bandage (AFB) was the foundation of hemorrhage control. A thick layer of cotton wrapped in layers of gauze was the simple premise to the AFB. This could be wrapped around an injured extremity to absorb blood as well as compress the wound. Since that time many hemostatic agents have been developed including the dry fibrin sealant dressing (American Red Cross Holland Laboratory, Rockville, MD, USA), Fibrin Patch (Ethicon, Inc., Somerville, NJ, USA), rapid deployment hemostat (Marine Polymer Technologies, Inc., Danvers, MA, USA), HemCon Bandage (Oregon Medical Laser Center, Portland, OR, USA), and QuikClot (Z-Medica Corp., Wallingford, CT, USA). The HemCon bandage consists of freeze-dried chitosan, which appears to strongly adhere to wet tissues and seal injured vessels. QuikClot functions as a mineral-based hemostatic agent. The zeolite minerals cause rapid water absorption which may concentrate clotting proteins and cells into the wound. The newer generation mineral-based hemostatic agents QuikClot Combat Gauze (Z-Medica Corp., Wallingford, CT, USA) and Celox (MedTrade Products Ltd., Crewe, UK) have shown improved hemostatic efficacy over previous topical hemostatic dressings (26).

Previously, efficacy and safety concerns arose with the HemCon and QuikClot dressings (27). Improvements in the current formulations of these dressings have since made them safer and more effective for clinical use (28–31).

Currently approved topical hemostatic agents can be categorized under four functional categories: biologically active agents, fibrin sealants, mechanical barrier agents, and flowable sealants (thrombin plus a mechanical barrier). Mechanical barriers block blood flow and create thrombogenic surfaces. QuikClot remains the most commonly used of this category (30). Biologically active agents are indicated for minor bleeding and oozing. Thrombin and fibrinogen–thrombin-containing agents do not depend on patients’ intrinsic clotting mechanism. Flowable sealants are delivered in a paste-like mixture and combine thrombin with a mechanical hemostatic agent. Fibrin sealants combine human fibrinogen with thrombin immediately before use and cause more rapid clot formation than a patient’s own coagulation cascade. Fibrinogen concentrations up to 25 times greater than physiologic concentrations may be produced at sites of hemorrhage (32).

The use of topical hemostatic agents in combination as an adjunct to surgical bleeding control has been identified as a grade 1B recommendation in European guidelines (17). Like many tenants of the care of patients with traumatic injuries, military experiences in the use of topical hemostatic agents have advanced the care of civilians.

## Principles of DC Surgery

Surgical intervention for DC in trauma involves an abbreviated exploratory laparotomy in an unstable patient. The triad of hypothermia, acidosis, and coagulopathy as well as shock physiology would lead to acute decompensation in these patients if not immediately intervened upon. Focused Assessment with Sonography for Trauma may be used in the trauma bay to aid in diagnosis of intraabdominal pathology. The DC laparotomy may be divided into three phases. Phase I is based on acute hemorrhage control, repair of visceral injuries using stapling methods, abdominal packing, and transport to an ICU. Phase II is based on aggressive resuscitation as described in previous sections. The basis of Phase III involves a return to the operating room within 72 h for removal of packing and reestablishment of intestinal continuity. While indications for DC laparotomy may differ among level 1 trauma centers, institutional algorithms are a mainstay of treatment in the trauma population (33, 34).

Patients with life-threatening hypotension and hypovolemia secondary to hemorrhage may require aortic occlusion to facilitate resuscitation. Aortic occlusion is thought to preserve coronary filling and cerebral perfusion in the setting of physiologic collapse due to hemorrhage although most of the data collected on clinical use have been limited and retrospective in nature (35–37). Traditionally, aortic occlusion was achieved by cross-clamping the descending thoracic aorta *via* thoracotomy; however, newer strategies such as the use of resuscitative endovascular balloon occlusion of the aorta (REBOA) have become more common in level 1 trauma centers. Use of REBOA has been shown to improve hemodynamics, increase survival as compared to historical controls who underwent thoracotomy (21.1% versus 7.4%), and preserve neurological outcome in survivors (38). Access site complications and limb-related adverse events remain low, and the use of smaller systems (7 Fr) may aid in increasing safety (39, 40). Increasing use of this strategy may require a shift in the training paradigm of acute care and trauma providers to include endovascular skills.

## Conclusion

Early management of vascular injury, starting in the field, is imperative for survival no less than any operative maneuver. Fluid resuscitation within the limits of permissive hypotension and appropriate use of tourniquets are part of a DCR concept that bond with DC surgery to guarantee optimal outcomes in contemporary high impact civilian trauma.

## Author Contributions

Substantial contributions to the conception or design of the work; or the acquisition, analysis, or interpretation of data for the work: EP, KS, EA, AA, AntreasP, and AnastasiaP. Drafting the work or revising it critically for important intellectual content: EP, KS, SG, and IK. Final approval of the version to be published: EP and EA.

## Conflict of Interest Statement

The authors declare that the research was conducted in the absence of any commercial or financial relationships that could be construed as a potential conflict of interest. The reviewer FS declared a shared affiliation, with no collaboration, with the authors. The reviewer JR and handling editor declared their shared affiliation.
